# Conspicuity and muscle-invasiveness assessment for bladder cancer using VI-RADS: a multi-reader, contrast-free MRI study to determine optimal b-values for diffusion-weighted imaging

**DOI:** 10.1007/s00261-022-03490-9

**Published:** 2022-03-18

**Authors:** Andrea Delli Pizzi, Domenico Mastrodicasa, Alessio Taraschi, Nicoletta Civitareale, Erica Mincuzzi, Stefano Censi, Michele Marchioni, Giulia Primiceri, Pietro Castellan, Roberto Castellucci, Giulio Cocco, Piero Chiacchiaretta, Antonella Colasante, Antonio Corvino, Luigi Schips, Massimo Caulo

**Affiliations:** 1 Department of Innovative Technologies in Medicine & Dentistry, “G. d’Annunzio” University, Chieti, Italy; 2 Department of Radiology, Stanford University School of Medicine, Stanford, CA USA; 3 Unit of Radiology, “Santissima Annunziata” Hospital, Chieti, Italy; 4 Department of Neuroscience, Imaging and Clinical Sciences, “G. d’Annunzio” University, Chieti, Italy; 5 Department of Medical, Oral and Biotechnological Sciences, G. d’Annunzio University of Chieti, Urology Unit, SS Annunziata Hospital, Chieti, Italy; 6 Laboratory of Biostatistics, Department of Medical, Oral and Biotechnological Sciences, “G. D’Annunzio” University, Chieti, Italy; 7 Unit of Ultrasound in Internal Medicine, Department of Medicine and Science of Aging, “G. D’Annunzio” University, Chieti, Italy; 8 Center of Advanced Studies and Technology (CAST), “G. d’Annunzio” University of Chieti-Pescara, Chieti, Italy; 9 Department of Psychological, Health and Territory Sciences, “G. d’Annunzio” University of Chieti-Pescara, Chieti, Italy; 10 Surgical Pathology Unit, SS Annunziata Hospital, Chieti, Italy; 11 Motor Science and Wellness Department, University of Naples “Parthenope”, Naples, Italy

**Keywords:** Bladder cancer, VI-RADS, MRI, Diffusion-weighted imaging

## Abstract

**Objective:**

To (1) compare bladder cancer (BC) muscle invasiveness among three b-values using a contrast-free approach based on Vesical Imaging-Reporting and Data System (VI-RADS), to (2) determine if muscle-invasiveness assessment is affected by the reader experience, and to (3) compare BC conspicuity among three b-values, qualitatively and quantitatively.

**Methods:**

Thirty-eight patients who underwent a bladder MRI on a 3.0-T scanner were enrolled. The gold standard was histopathology report following transurethral resection of BC. Three sets of images, including T2w and different *b*-values for DWI, set 1 (*b* = 1000 s/mm^2^), set 2 (*b* = 1500 s/mm^2^), and set 3 (*b* = 2000 s/mm^2^), were reviewed by three differently experienced readers. Descriptive statistics and Intraclass Correlation Coefficient (ICC) were calculated. Comparisons among readers and DWI sets were performed with the Wilcoxon test. Receiver operating characteristic (ROC) analysis was performed. Areas under the curves (AUCs) and pairwise comparison were calculated.

**Results:**

AUCs of muscle-invasiveness assessment ranged from 0.896 to 0.984 (reader 1), 0.952–0.968 (reader 2), and 0.952–0.984 (reader 3) without significant differences among different sets and readers (*p* > 0.05). The mean conspicuity qualitative scores were higher in Set 1 (2.21–2.33), followed by Set 2 (2–2.16) and Set 3 (1.82–2.14). The quantitative conspicuity assessment showed that mean normalized intensity of tumor was significantly higher in Set 2 (4.217–4.737) than in Set 1 (3.923–4.492) and Set 3 (3.833–3.992) (*p* < 0.05).

**Conclusion:**

Muscle invasiveness can be assessed with high accuracy using a contrast-free protocol with T2W and DWI, regardless of reader’s experience. *b* = 1500 s/mm^2^ showed the best tumor delineation, while *b* = 1000 s/mm^2^ allowed for better tumor–wall interface assessment.

## Introduction

Bladder cancer (BC) is a leading cause of cancer-related death in men and accounts for approximately 550.000 new cancer cases per year worldwide [1]. Muscle-invasive BC (MIBC) represents about a quarter of the total and is > T1 tumor (growth into the muscle layer). MIBC usually requires radical cystectomy, with or without neo- or adjuvant chemotherapy, thus significantly impacting patients’ survival [2]. Non-muscle-invasive BC (NMIBC) (Ta–T1 tumor) may usually benefit from local treatments and has a better prognosis [3]. Accurate preoperative staging of bladder cancer is essential in determining the extent of disease and optimal treatment. Magnetic resonance imaging has recently gained traction due to its accuracy for the local staging and the assessment of muscle invasiveness [4–9]. This is largely due to the combination of high-resolution anatomical sequence (T2-weighted images—T2W) with functional sequences (Diffusion-Weighed Imaging—DWI, and Dynamic Contrast Enhanced—DCE), especially when using high field strength (3.0-T) [4, 10, 11]. In 2018, Vesical Imaging-Reporting And Data System (VI-RADS) introduced a standardized reporting criterion for bladder MRI to improve communication among doctors and facilitate patient management [5]. Several studies demonstrated high accuracy of VI-RADS for discriminating MIBC and NMIBC [12–16]. The standard MR protocol is currently defined “three-parametric” and includes conventional T2W images, DWI, and DCE imaging. Among these sequences, DWI has been considered the most relevant sequence to estimate muscle invasion [5]. A study investigating the feasibility of contrast-free MR imaging was recently proposed [17]. The authors reported that the diagnostic accuracy of a contrast-free MR imaging protocol, including only T2w and DWI, was comparable to the standard three-parametric protocol for the detection of MIBC, regardless of the reader experience. However, to the best of our knowledge, the best diffusion gradient strengths (*b*-values) to evaluate muscle invasiveness and tumor conspicuity have yet to be defined. Based on recent studies assessing different tumor type conspicuity using different diffusion gradient strengths, two are the most relevant issues [18–25]. On the one hand, a high diffusion gradient strength would provide a better background suppression, thus allowing an easier delineation of the tumor. On the other hand, it may result in a reduced signal-to-noise ratio with a subsequent overall decreased signal intensity and anatomical detail [26]. This approach may have a twofold beneficial effect. First, considering the emerging role of VI-RADS, it may help to optimize the standard MR imaging protocol. Second, in a contrast-free MR imaging setting, it may have a beneficial impact on costs, scan time, and patient safety, especially considering the potential future extension of VI-RADS to the surveillance and treatment response assessment [27, 28]. For these reasons, in this proof-of-concept study, we aimed to (1) compare the muscle invasiveness of tumor among three different *b*-values using a VI-RADS-based contrast-free approach, to (2) determine if the muscle-invasiveness assessment is affected by the reader experience, and to (3) compare the conspicuity of bladder cancer among three different *b*-values both qualitatively and quantitatively.

## Material and methods

### Patient population and study design

An Institutional Review Board and Ethical Committee approval were received for this prospective study. The study was performed in line with the European Urology and Good Clinical Practice guidelines and conducted accordingly to ethical principles laid down by the latest version of the Declaration of Helsinki [7]. Written informed consent was obtained from all patients enrolled in the study. A total of 41 patients who underwent multiparametric-MRI (mp-MRI) between August 2019 and December 2019 were prospectively included. The study population was previously described in detail [16, 17]. For this study the inclusion criteria were 1) endoscopic findings suggestive of BC, 2) MRI of bladder performed on a 3-T scanner, and 3) TURB with histological evaluation. Finally, three patients were excluded: two patients for severe artifacts in the pelvis (one patient showed susceptibility artifacts due to hip replacement and one patient showed motion artifacts on DWI) and one patient was scanned on a 1.5 T scanner. The final study population was composed of 38 patients.

### MRI protocol

A state-of-the-art 3-T MR scanner (dStream, Philips Medical System, Best, the Netherlands) equipped with a phased array surface coil was used for all patients included in the study. The MR protocol included the following sequences: T2W turbo spin-echo images, DWI (including *b*=1000 s/mm^2^, *b* = 1500 s/mm^2^, *b* = 2000 s/mm^2^) and DCE T1-weighted 3D spoiled gradient-echo images (DCE). A more detailed description of the MR parameters is reported in Table 1. Apparent diffusion coefficient maps (ADC maps) were calculated for each patient. Gadoteridol (Prohance; Bracco, Milan, Italy) was used in a dose of 0.2 mmol/kg (flow rate of 2 mL/s). Patients received 20 mg of scopolamine butylbromide (Buscopan, Boehringer Ingelheim, Ingelheim am Rhein, Germany) intravenously to reduce the incidence of motion artifacts due to bowel motility. Patients were instructed to void 1–2 h before imaging and started drinking 500–1000 ml of water 30 min before the examination [5]. The degree of bladder filling was evaluated using ultrasound before patient entered the MR room.

**Table 1:** Parameters of T2-weighted and DWI sequences included in the three sets of images

	Set 1	Set 2	Set 3	Set 1	Set 2	Set 3
	T2cb-weighted Fast-Spin Echo			Diffusion-Weighted Imaging (DWI)*		
Repetition time (ms)	3000–5000			3000		
Echo time (ms)	80			97		
Matrix	200 × 179			68 × 54		
Flip angle	90			90		
Number of excitations	2			3–12		
Section thickness (mm)	4			4		
*b*-value ( s/mm^2^)	–			1000	1500	2000
Imaging planes	Transverse^†^, Coronal, Sagittal			Transverse^†^, Sagittal		
Acquisition time (min)	2.26			4.19		

DWI sequences included ADC map calculation

^*^DWI performed with *b*-values of 0, 600, 1000, 1500, and 2000 s/mm

^†^Transverse plane angulated perpendicularly to the long axis of the bladder

**Table 2:** Descriptive baseline characteristics of included patients (*n* = 38)

Features	Value
Age	72.5 (66.5—81.0)
Gender, Male	27 (71.4%)
Body mass index, kg/m^2^	26.6 (24.0—29.1)
*Charlson Comorbidity Index*	
0	21 (55.3%)
1	13 (34.2%)
2	3 (7.9%)
3	1 (2.6%)
*Urine cytology*	
Non-diagnostic	13 (34.2%)
Negative	16 (42.1%)
Positive	9 (23.7%)
*Previous endovescical treatment*	
Bacillus Calmette-Guerin	4 (10.5%)
Epirubicin	1 (2.6%)
Mitomycin C	3 (7.9%)
None	30 (78.9%)

Continuous variables are presented as median and interquartile ranges (IQR). Categorical variables are presented as frequencies and percentages (%)

### Image analysis

Three sets of images, namely set 1, set 2, and set 3, were evaluated on a dedicated workstation. Each set included axial, sagittal, and coronal T2W images and DWI images with the corresponding ADC map. In detail, *b* = 1000 s/mm^2^ images were included in set 1, *b* = 1500 s/mm^2^ images in Set 2, and *b* = 2000 s/mm^2^ in set 3. Each set of images included axial and sagittal planes and was randomly assigned to three readers with different levels of experience in abdominal radiology (1 radiologist with 10 years of expertise in abdominal MRI, one senior resident with two years of expertise in abdominal MRI, and one second-year resident with 1 year of expertise in abdominal MRI) in three separate reading sessions with at least a month in between, to avoid recall bias (Table 2).

### Muscle-invasiveness assessment

MRI criteria for muscle invasiveness were assessed according to VI-RADS as well as the bladder subdivision in sectors [5, 16]. In this way, twelve sectors were considered. Following VI-RADS, all detected lesions were scored on a 5-point scale based on the likelihood of muscle invasion: 1, highly unlikely; 2, unlikely; 3, equivocal; 4, likely; and 5, very likely [5].

### Qualitative conspicuity assessment

**Fig. 1:**
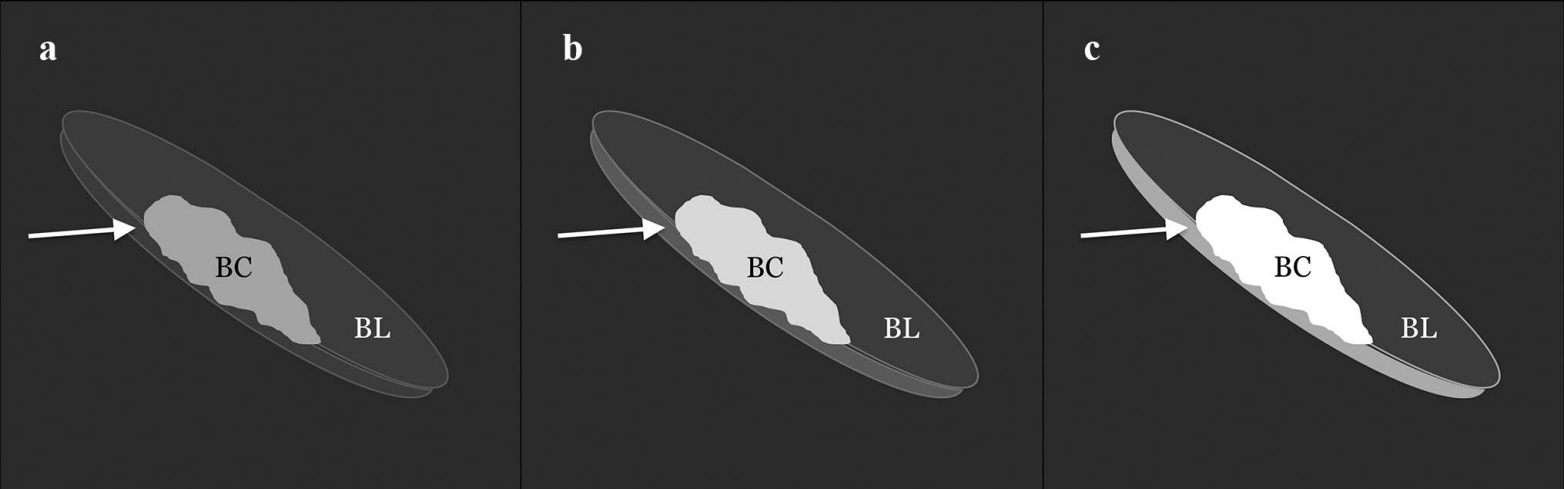
Three-point scale used to assess tumor conspicuity on DWI: **a** hyperintense lesion, but only slightly demarcated from the background and/or poor tumor–wall interface, **b** hyperintense lesion, well demarcated from the background with good tumor–wall interface, and **c** hyperintense lesion with excellent background suppression and optimal tumor–wall interface. BC = bladder cancer; arrow = bladder wall; BL = bladder lumen

The three readers independently qualitatively assessed 3 sets of images. In detail, they scored the tumor conspicuity on DWI using a 3-point scale: (1) hyperintense lesion, but only slightly demarcated from the background and/or poor tumor–wall interface, (2) hyperintense lesion, well demarcated from the background with good tumor–wall interface, and (3) hyperintense lesion with excellent background suppression and optimal tumor–wall interface (Fig. 1). The T2w images were available to the readers for anatomical reference.

### Quantitative conspicuity assessment

**Fig. 2:**
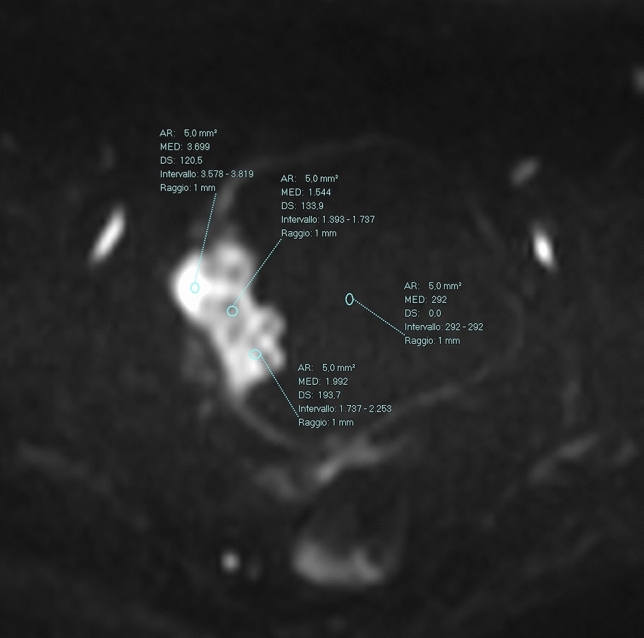
Quantitative analysis. Three circular 5-mm^2^ regions of interest (ROIs) of the tumor were placed on DWI images. Another circular 5-mm^2^ ROI was also placed in the bladder content to normalize data. Tumor conspicuity was defined as the ratio between the mean tumor intensity and the bladder content intensity

To quantitatively assess the tumor conspicuity, the expert reader used a circular 5-mm^2^ region of interest (ROI) of the tumor on DWI images using T2-weighted images for anatomical reference (Fig. 2). In detail, a single-slice measurement was performed by placing three ROIs randomly in the tumor and calculating the mean tumor intensity. A circular 5-mm^2^ region of interest was also placed in the bladder content to normalize data. Tumor conspicuity was defined as the ratio between the mean tumor intensity and the bladder content intensity.

## Reference standard

Urologic evaluation included the description of lesion number, size, morphology, and location. A standardized form with a descriptive schematic map was used for cystoscopy, MRI, and TURB, to record all the information on each lesion [16]. All patients underwent a standard TURB and a piecemeal resection in fraction. The base of each lesion was sent to histological evaluation separately with a numerical code to compare histological results with those of cystoscopy and mp-MRI. When indicated, a second TURB was performed according to the EAU guidelines [7]. Specimens were examined to assess the type, grade, and stage of the tumor. The World Health Organization classification was used to classify and grade malignant tumors, while the American Joint Committee on Cancer/Union for International Cancer Control TNM system was used to stage each lesion [7].

## Statistical analysis

Sample size estimation was performed considering the association between VI-RADS score and MIBC status. The reference was the proportion of NMIBC and MIBC stratified according to VI-RADS score in previous studies [16, 29]. A power of 90% and a value of 5% were considered for the *χ*^2^ test. Sample size was estimated using the R package “pwr” (version 1.2.2; function: pwr.chisq.test). The estimated effect size was 0.7, and the number of patients needed to obtain the desired power was 31 subjects. A dichotomization of VI-RADS scores was performed. In detail, concerning the diagnostic accuracy for the detection of MIBC, VI-RADS scores of 1–3 were considered negative, while VI-RADS scores of 4–5 were considered positive. Descriptive statistics and Intraclass Correlation Coefficient (ICC) were calculated for each reader. Comparison among readers and sets was achieved with Wilcoxon test. Receiver operating characteristic (ROC) analysis was performed, and areas under the curve (AUCs) were calculated for each reader and image set. ROC curves were compared to test the difference between the areas under the ROC curves among the three readers. ROC curve comparison was performed with MedCalc software, version 16.8.4 (MedCalc Software, Ostend, Belgium). All other statistical analyses were performed using IBM SPSS Statistics software, version 20 (IBM, Armonk, NY). A *p*-value ≤ 0.05 was considered statistically significant.

## Results

Twenty-seven patients of the study were male (27, 71%) with a median age of 72.5 (IQR 66.5–81.0) years. Out of the 38 patients, 31 (82%) had a NMBIC (Ta–T1) and 7 (18%) had a MIBC (T2–T3). Sixty-eight BCs were diagnosed of which 33 (48.5%) were Ta (non-invasive papillary carcinoma), 28 (41.2%) were T1 (subepithelial connective tissue invasion), 6 (8.8%) were T2 (muscle invasion), and 1 (1.5%) was T3 (perivesical tissue invasion) [30]. The mean dimension, measured at cystoscopy, of NMIBC was 9.8 mm (range 3–40 mm), while the mean dimension of MIBC was 29 mm (range 10–50 mm).

## Muscle-invasiveness assessment

**Fig. 3:**
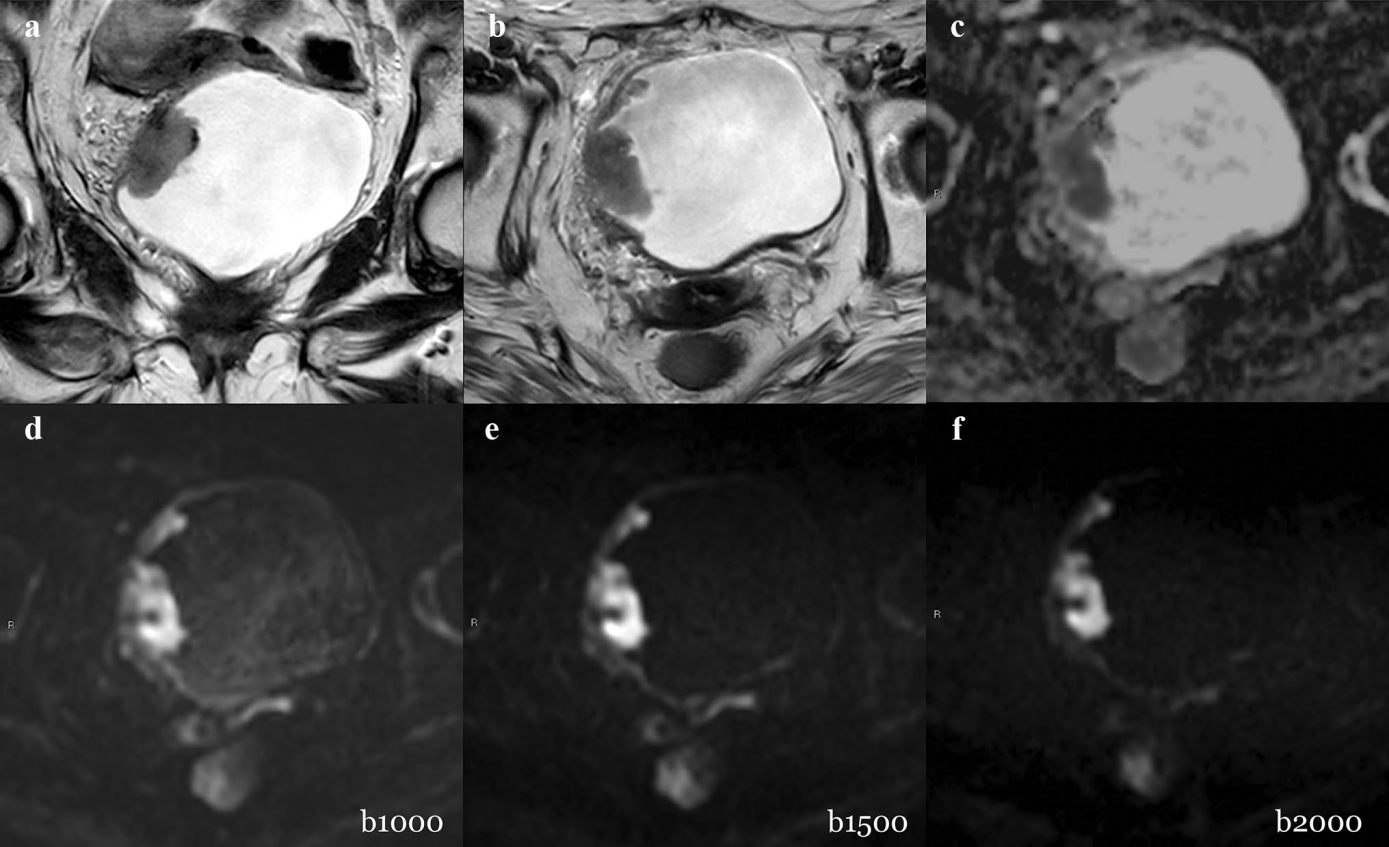
Example of muscle-invasive BC classified correctly. A 79-year-old woman with hematuria and a bladder mass reported after flexible cystoscopy underwent bladder MRI before primary TURB. Coronal (**a**) and axial (**b**) T2W imaging showed a thickened right-lateral wall. All gradient strengths (b1000, b1500, and b2000) and ADC map (**c**) confirmed the restricted diffusion of the tumor extending to the muscular layer. The tumor conspicuity on b1000 (**d**) was higher than b1500 (**e**) and b2000 (**f**) due to better tumor–wall interface visualization. The T stage after TURB was HG-T2 (TURB). DWI = diffusion-weighted imaging; HG = high grade; MRI = magnetic resonance imaging; T2W = T2 weighted; TURB = transurethral resection of the bladder; VI-RADS = Vesical Imaging-Reporting and Data System

**Fig. 4:**
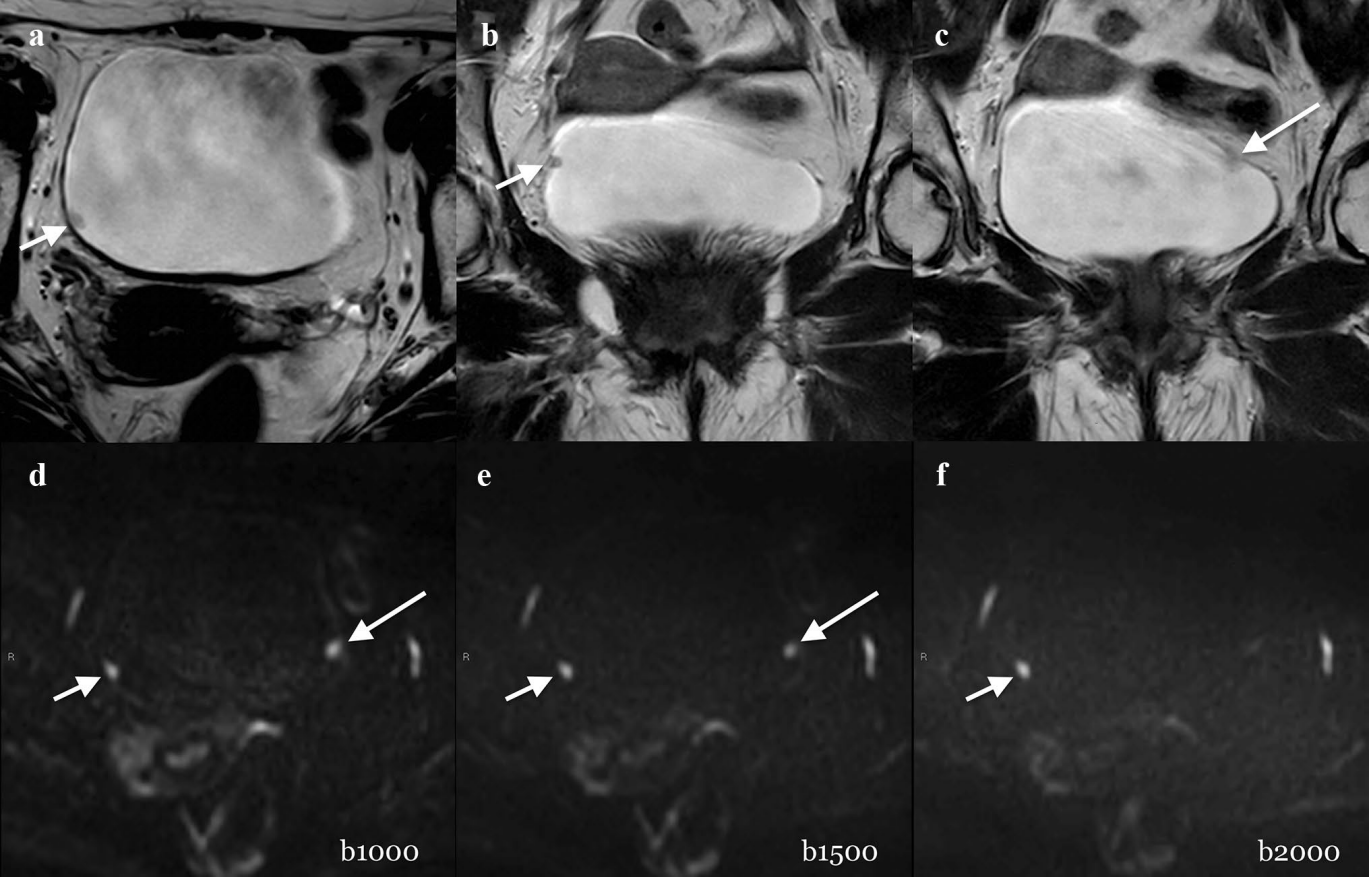
Example of a not muscle-invasive BC classified incorrectly. A 73-year-old man with hematuria and two polyps, documented after flexible cystoscopy, underwent MRI before primary TURB. Axial (**a**) and coronal (**b**, **c**) T2W imaging (T2) showed a small (4 mm) polypoid lesion on the right wall of the bladder (short arrow in **a** and **b**). The lesion was well detected by the three readers regardless the image set and was scored as VI-RADS 1 (short arrow in **d**, **e,** and **f**). Another slightly visible small (4 mm) non-muscular invasive lesion (VI-RADS 1) on the left wall of the bladder was suspected on T2 images (long arrow in **c**). However, it was definitely detected by the three readers only when reading b1000 and b1500 images (long arrow in **d** and **e**), but not on b2000 images. T stage after TURB was LG-T1 (TURB). After four weeks, Re-TURB was performed and it confirmed the absence of residual tumor. DWI = diffusion-weighted imaging; HG = high grade; MRI = magnetic resonance imaging; T2W = T2 weighted; TURB = transurethral resection of the bladder; VI-RADS = Vesical Imaging-Reporting and Data System

The 7 MIBCs were correctly identified by all readers using every set of images. Table 3 shows the diagnostic performance of the readers for the MIBC detection. In detail, no significant differences were observed in diagnostic performance for all readers among the 3 sets of images (*p* > 0.05). The AUCs for the three sets of images ranged from 0.896 to 0.984 (reader 1), 0.952 to 0.968 (reader 2), and 0.952 to 0.984 (reader 3), respectively. No significant differences in diagnostic performance were found among the three readers in the pairwise comparison (*p* > 0.05). Table 4 shows a per-lesion diagnostic performance analysis for each reader to classify BCs according to the TNM classification. False-positive cases occurred only in NMIBCs, mostly in set 1 and set 2 (7 and 10 vs. 5 of Set 3). Out of 114 false negatives, extracted from all readers, 113 were NMIBCs. False-negative cases increased for all three readers when using Set 3 (from 12 to 17 for reader 1, from 10 to 12 the reader 2, and from 13 to 14 for reader 3). Case examples of correctly and incorrectly classified MIBC are shown in Figs. 3 and 4, respectively.

**Table 3:** Diagnostic performance of the three readers regarding the MIBC detection for Set 1, Set 2, and Set 3

	AUC MIBC vs. NMIBC (Standard error)			Pairwise readers	*p*-value
	Set 1	Set 2	Set 3		
*Reader 1*	0.896 (0.075)	0.968 (0.022)	0.984 (0.016)	Reader 1 vs Reader 2 Reader 1 vs Reader 3 Reader 3 vs Reader 2	> 0.05 > 0.05 > 0.05
*Reader 2*	0.968 (0.022)	0.952 (0.027)	0.968 (0.022)		
*Reader 3*	0.952 (0.027)	0.952 (0.027)	0.984 (0.016)

**Table 4:** Per-lesion diagnostic performance of the three readers to correctly classify BCs according to TNM Stage Classification

		Set 1			Set 2			Set 3		
		Correctly Classified	Incorrectly Classified		Correctly Classified	Incorrectly Classified		Correctly Classified	Incorrectly Classified	
			False Negatives	False Positives		False Negatives	False Positives		False Negatives	False Positives
Ta (*n* = 33)	*Reader 1*	22	11	0	20	11	2	19	14	0
	*Reader 2*	25	8	0	24	8	1	23	10	0
	*Reader 3*	20	11	2	20	11	2	20	12	1
T1 (*n* = 28)	*Reader 1*	24	2	2	26	1	1	24	3	1
	*Reader 2*	24	2	2	24	2	2	24	2	2
	*Reader 3*	25	2	1	24	2	2	25	2	1
T2 (*n* = 6)	*Reader 1*	5	1	0	6	0	0	6	0	0
	*Reader 2*	6	0	0	6	0	0	6	0	0
	*Reader 3*	6	0	0	6	0	0	6	0	0
T3 (*n* = 1)	*Reader 1*	1	0	0	1	0	0	1	0	0
	*Reader 2*	1	0	0	1	0	0	1	0	0
	*Reader 3*	1	0	0	1	0	0	1	0	0

## Qualitative conspicuity

Results of the qualitative conspicuity assessment are shown in Table 5. The mean conspicuity scores were higher in Set 1 (2.21, 2.31, and 2.33 for reader 1, 2, and 3, respectively), followed by Set 2 (2, 2.16, and 2.15 for reader 1, 2, and 3, respectively) and Set 3 (2.14, 1.82, and 1.91 for reader 1, 2, and 3, respectively) for all readers. The ICC among readers was 0.75 (set 1), 0.77 (set 2), and 0.81 (set 3).

**Table 5:** Qualitative assessment of tumor conspicuity for Set 1, Set 2, and Set 3 based on a 3-point scale

	Qualitative Assessment Mean Tumor Conspicuity (Standard Deviation)			ICC (95% Confidence Intervall)		
	Set 1	Set 2	Set 3	Set 1	Set 2	Set 3
*Reader 1*	2.31 (0.69)	2.00 (0.76)	2.14 (0.87)	0.75 (0.60–0.85)	0.77 (0.64–0.86)	0.81 (0.70–0.89)
*Reader 2*	2.21 (0.77)	2.16 (0.75)	1.82 (0.79)			
*Reader 3*	2.33 (0.75)	2.15 (0.78)	1.91 (0.88)			

## Quantitative conspicuity

The mean normalized intensity of the tumor was significantly higher when using Set 2 (4.737, 4.217, and 4.608 for reader 1, 2, and 3, respectively) compared to Set 1 (4.123, 3.923, and 4.492 for reader 1, 2, and 3, respectively) and Set 3 (3.896, 3.992, and 3.833 for reader 1, 2, and 3, respectively). The ICC among readers was 0.79 (set 1), 0.83 (set 2), and 0.91 (set 3). Results of the quantitative conspicuity assessment and pairwise comparison are shown in Table 6.

**Table 6:** Quantitative assessment of tumor conspicuity for Set 1, Set 2, and Set 3

	Quantitative Assessment													
	Tumor Intensity* (Standard Deviation)			Bladder Content Intensity* (Standard Deviation)			Tumor Conspicuity* (Standard Deviation)			ICC (95% CI**)			Pairwise Comparison	p-value***
	Set 1	Set 2	Set 3	Set 1	Set 2	Set 3	Set 1	Set 2	Set 3	Set 1	Set 2	Set 3		
*Reader 1*	1903.36 (741.70)	1907.11 (779.04)	1765.49 (759.19)	562.40 (246.47)	431.09 (122.31)	499.63 (146.01)	4.12 (2.72)	4.74 (2.31)	3.90 (2,29)	0.79 (0.67–0.87)	0.83 (0.74–0.90)	0.91 (0.85–0.94)	Set 1 vs. Set 2 Set 1 vs. Set 3 Set 3 vs. Set 2	0.012 1.000 0.001
*Reader 2*	2000.21 (786.60)	1991.77 (789.82)	1799.52 (787.77)	572.58 (200.41)	540.92 (203.88)	517.86 (181.72)	3.92 (2.17)	4.22 (2.41)	3.99 (2.70)					
*Reader 3*	2083.71 (675.69)	1883.91 (657.04)	1762.44 (697.06)	543.22 (222.29)	434.34 (109.86)	505.44 (159.99)	4.49 (2.30)	4.61 (1.97)	3.83 (1.98)					

^*^Mean value

^**^Confidence Interval

^***^Wilcoxon Test with Bonferroni’s correction

## Discussion

Our results showed that all *b*-values included in this study accurately assessed bladder cancer muscle invasiveness using a bi-parametric MR protocol. Moreover, the reader’s experience did not significantly affect the diagnostic accuracy. Our results confirmed the promising results of recent studies on the feasibility of contrast-free MRI protocols based on DWI [5, 17, 31]. Additionally, since they are almost comparable among the different readers, they also support the good VI-RADS repeatability even in less experienced readers [13]. Interestingly, if, on the one hand, muscle-invasiveness assessment was not significantly different among the three sets of images, the conspicuity varied both qualitatively and quantitatively based on different *b*-values. While the best qualitative scores were obtained with set 1, corresponding to the lowest *b*-value (*b* = 1000 s/mm^2^), set 2 led to the best quantitative scores corresponding to intermediate–high *b*-value (*b* = 1500 s/mm^2^). Set 3, which included the highest *b*-value (*b* = 2000 s/mm^2^), showed the lowest qualitative scores and signal intensity at the quantitative analysis. We believe that there are two possible explanations. First, from a quantitative perspective, the reduced signal intensity in the bladder content at higher *b*-values resulted in higher tumor conspicuity: the higher background suppression facilitated the tumor visualization. However, the lower intensity in the bladder content corresponded to a reduction of the mean tumor signal intensity compared to lower *b*-values. For this reason, the mean quantitative scores at the highest gradient strengths resulted in low signal intensity of the tumor. In our study, Set 2 resulted in the highest tumor signal intensity, providing the best ratio of background suppression and signal-to-noise reduction. To this end, we recommend using a high-gradient strength DWI using an adequate number of signal average and repetition time to balance the signal-to-noise ratio reduction [18].

Second, to explain the results of qualitative assessment, the use of high *b*-values is burdened with the overall reduced signal, which translates into poor anatomical visualization. The loss of potentially valuable information could thus reduce the diagnostic confidence of the reader. This may be even more evident when an adequate anatomical detail is required, for example, in the bladder wall assessment or in the case of small subtle lesions. In fact, the per-lesion analysis showed the link between the highest gradient strength (*b* = 2000 s/mm^2^) and the increased number of false-negative cases compared to the other sets of images. Most of these cases were due to small (< 5 mm) lesions with substantial overlap among the readers, ranging from 70 to 75%.

To the best of our knowledge, this is the first study comparing the conspicuity and the muscle-invasiveness assessment of different contrast-free bladder MRI settings using VI-RADS in a clinical scenario. By supporting recent studies on the optimization of bladder MRI and the promising results regarding the feasibility of a contrast-free MRI protocol, our results may have a beneficial effect on patient safety costs, scan time, and patient safety [31–33].

Our study has some limitations. First, ours is a single-center study with a relatively low number of patients. Nonetheless, our investigation was intended as a proof-of-concept study. Larger studies, possibly including multiple institutions, are needed to validate our results. Second, the type of VI-RADS scores dichotomization may have represented a selection bias. In fact, the appropriate VI-RADS cut-off defining whether the tumor is muscle invasive is controversial [16, 34]. According to our methodology, Marchioni et al. recently demonstrated that a threshold of 4 significantly improved MIBC detection reaching an accuracy of 90% [16]. Third, the physician experience and the patient characteristics influenced the patient selection. For example, patients with worrisome tumor features (i.e., large and solid tumors) may not have been included to avoid delaying active treatment. Fourth, a degree of unbalanced data (MIBC vs. NMIBC) was present, which could have affected our results. However, considering that our primary aim was to investigate if any difference in terms of conspicuity and muscle-invasiveness assessment among different contrast-free MRI settings exists, we adopted a multi-reader approach to improve the generalization of our results. Moreover, both per-patient and per-lesion analyses were performed for each reader to test different clinical settings.

## Conclusion

Bladder cancer muscle invasiveness can be accurately assessed using a bi-parametric MRI protocol consisting of T2w and DWI, regardless of the diffusion gradient strengths (1000–2000 s/mm^2^) and the reader’s experience. Intermediate–high *b*-value (*b* = 1500 s/mm^2^) showed the highest signal intensity due to an adequate balance between background suppression and signal-to-noise reduction. A *b*-value of 1000 s/mm^2^ allowed better tumor–wall interface assessment. Very high *b*-value (*b* = 2000 s/mm^2^) was associated with lower conspicuity and increased false-negative cases, especially for small (< 5 mm) lesions. Further validation studies are warranted to define an optimization of the current bladder MRI protocol and to evaluate the potential clinical role of a contrast-free MRI protocol.

## Abbreviations

ADC: Apparent diffusion coefficient
BC: Bladder cancer
MRI: Magnetic resonance imaging
DCE: Dynamic contrast enhanced
DWI: Diffusion-weighted imaging
MIBC: Muscle-invasive bladder cancer
NMIBC: Non-muscle-invasive BC
T2W: T2W-weighted images
TURB: Transurethral resection of the bladder
VI-RADS: Vesical imaging-reporting and data system

## Declarations

### Guarantor

The scientific guarantor of this publication is Andrea Delli Pizzi.

### Statistics and biometry

No complex statistical methods were necessary for this paper.

### Study subjects or cohorts overlap

The study subjects have been previously reported in the studies: Marchioni, M. et al., Could Bladder Multiparametric MRI Be Introduced in Routine Clinical Practice? Role of the New VI-RADS Score: Results From a Prospective Study. Clin Genitourin Cancer, 2020. Delli Pizzi, A., Mastrodicasa, D., and Marchioni, M. et al. Bladder cancer: Do we need contrast injection for MRI assessment of muscle invasion? A prospective multi-reader VI-RADS approach. Eur Radiol 31, 3874–3883 (2021). https://doi.org/10.1007/s00330-020-07473-6
